# A Treatise on Emotional Disorders of the Sympathetic System of Nerves

**Published:** 1867-10

**Authors:** 


A Treatise on Emotional Disorders of the Sympathetic System of Nerves. By WILLIAMS MURRAY, M.D., M.R.C.P. Lond., Physician to the Dispensary and Hospital for Sick Children, and Lecturer on Physiology in the College of Medicine, Newcastle-on-Tyne. New York: A. SIMPSON & Co., 60 Duane Street. 1867.

This is a monograph of 95 octavo pages, printed on good type and paper. The work is divided into two sections. The first relates to the physiology of mental emotions and their effects on the various bodily functions; and the second to emotional diseases and their treatment. The subjects discussed are worthy of more consideration than is generally bestowed on them, and the work before us will fully repay the practitioner for a careful perusal.

For sale by S. C. GRIGGS & Co., 39 and 41 Lake St., Chicago. Price $1.50.

